# Supplementary material for: Targeting mutant p53-expressing tumours with a T cell receptor-like antibody specific for a wild-type antigen
Source: Nat Commun. 2019 Nov 26;10:5382. doi: 10.1038/s41467-019-13305-z (PMC6879612; doi:10.1038/s41467-019-13305-z)
Supplement: Supplementary file 1 — Supplementary Information [file 41467_2019_13305_MOESM1_ESM.pdf]

# Supplementary Information for

# Targeting Mutant p53-Expressing Tumours with a T Cell Receptor-like Antibody Specific for a Wild-type Antigen

Low *et al*

| Organs | Untreated | P1C1PNU-01 | P1C1PNU-02 |
|---|---|---|---|
| Brain | - NSF | - NSF | - NSF |
| Kidneys | - NSF | - NSF | - NSF |
| Thymus | - NSF | - Minimal (1) lympholysis | - NSF |
| Heart | - NSF | - NSF | - NSF |
| Liver | - Mild multifocal polymorphonuclear, mononuclear cell infiltrate with individual cell necrosis<br>- Moderate diffuse hepatocellular vacuolation | - Marked multifocal polymorphonuclear, mononuclear cell infiltrate with individual cell necrosis<br>- Moderate diffuse hepatocellular vacuolation | - Minimal multifocal, subcapsular, hepatocellular necrosis<br>- Minimal multifocal polymorphonuclear, mononuclear cell infiltrate<br>- Moderate diffuse hepatocellular vacuolation |
| Gallbladder | - NSF | - NSF | - NSF |
| Spleen | - NSF | - Mild lympholysis<br>- Mild increased hemosiderin aggregates | - Mild lympholysis<br>- Mild increased hemosiderin aggregates |
| Lung | - NSF | - NSF | - NSF |
| Small intestine | - NSF | - NSF | - NSF |
| Bone marrow | - NSF | - NSF | - NSF |

**Supplementary Table 1.** Summary of histopathological examination of HLA-A24 trangenic mice treated with P1C1TM-PNU. Hematoxylin and eosin stained fixed tissues of treated and control mice were assessed for treatment-associated pathology. NSF no significant findings

**a**

| Heavy Chain | | | | | | | | | | | | | | | | | | | | |
|---|---|---|---|---|---|---|---|---|---|---|---|---|---|---|---|---|---|---|---|---|
| **Residue** | 1 | 2 | 3 | 4 | 5 | 21 | 22 | 23 | 24 | 25 | 40 | 41 | 42 | 43 | 44 | 68 | 69 | 70 | 71 | 72 |
| **Original Seq.** | Q | L | Q | L | Q | T | C | A | V | S | R | Q | R | P | G | R | L | T | I | S |
| **Germline Seq.** | Q | V | Q | L | Q | T | C | T | V | S | R | Q | H | P | G | R | V | T | I | S |

| Light Chain | | | | | | | | | | | | | | | | | | | | |
|---|---|---|---|---|---|---|---|---|---|---|---|---|---|---|---|---|---|---|---|---|
| **Residue** | 1 | 2 | 3 | 4 | 5 | 6 | 7 | 8 | 9 | 38 | 39 | 40 | 41 | 42 | 60 | 61 | 62 | 63 | 64 |
| **Original Seq.** | Q | A | V | L | T | Q | P | S | S | Y | Q | H | L | P | V | P | H | R | F |
| **Germline Seq.** | Q | S | V | L | T | Q | P | P | S | Y | Q | Q | L | P | V | P | D | R | V |

**b**

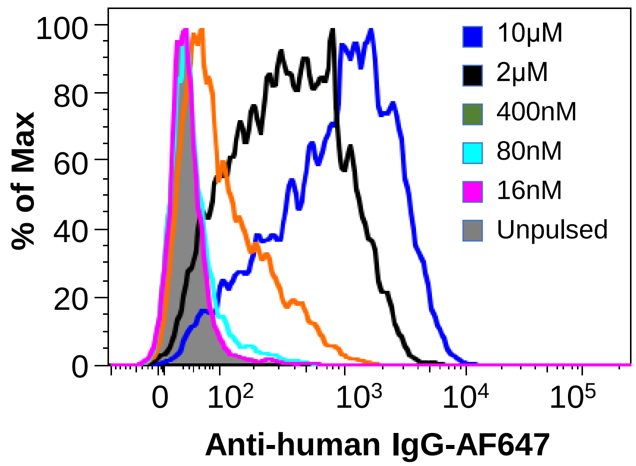


**Supplementary Fig. 1.** Conversion of TCR-like antibody P1C1 to germ line (P1C1gl) sequence. (A) Sequence alignment of P1C1 heavy chain and light chain with germ line IGHV4-31*03 and IGLV1-40*01 sequences. Amino acids that vary are labeled in red. **(B)** SaoS2 cells were pulsed with a range of concentrations of $p53_{125-134}$ peptides and stained with P1C1gl at 10μg $mL^{-1}$. Data is representation of three experiments.

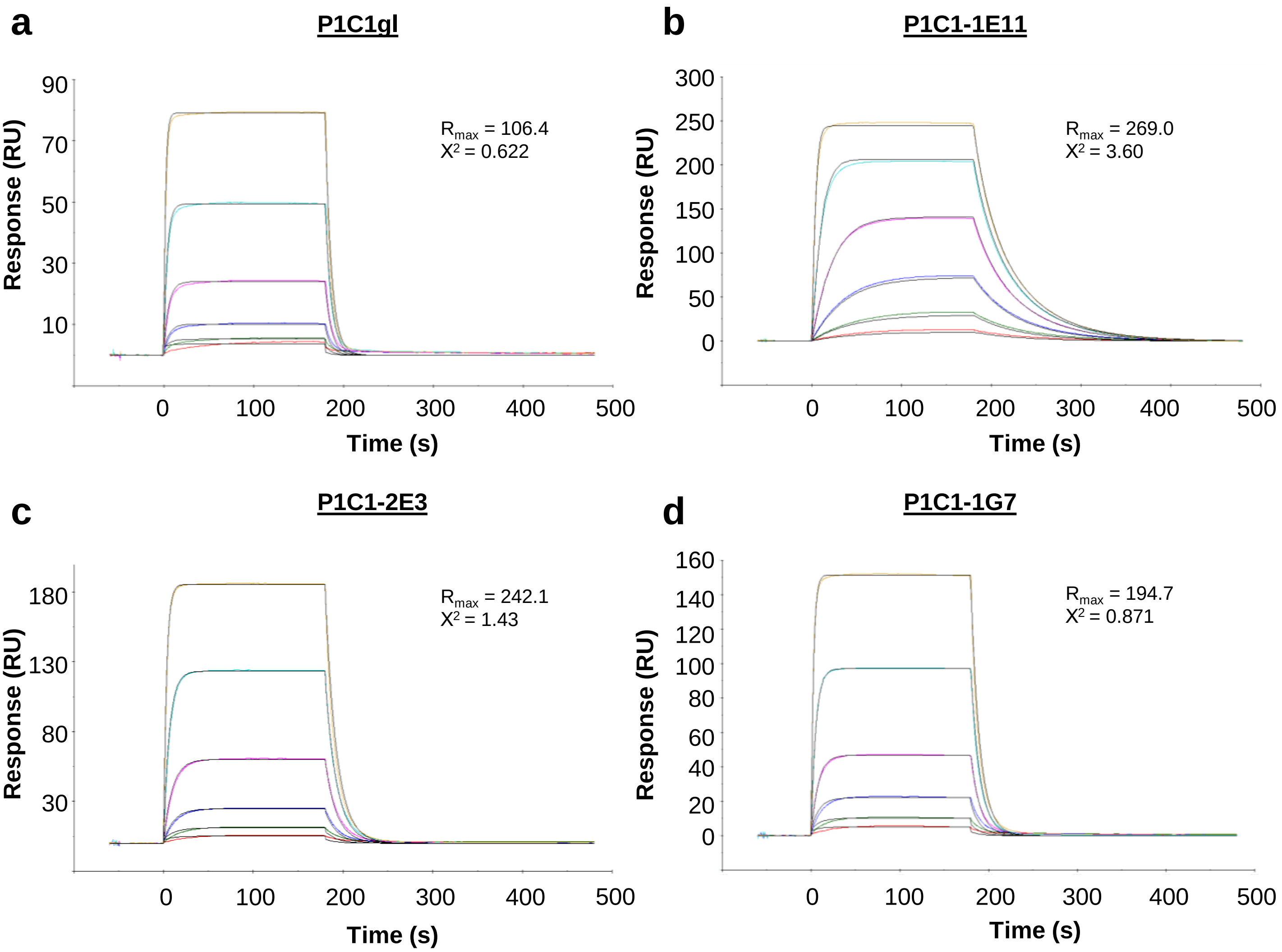


**e**

| | $k_a$/ x10$^6$ $M^{-1}s^{-1}$ | $k_d$/ x10$^{-2}$ $s^{-1}$ | $K_D$/nM |
|---|---|---|---|
| **P1C1gl** | 1.26 ± 0.189 | 13.94 ± 0.444 | 116 ± 13.4 |
| **P1C1-1E11** | 1.79 ± 0.0575 | 3.62 ± 0.0495 | 20.3 ± 0.360 |
| **P1C1-2E3** | 1.10 ± 0.0495 | 7.58 ± 0.270 | 69.2 ± 0.690 |
| **P1C1-1G7** | 1.48 ± 0.0830 | 9.72 ± 0.141 | 66.0 ± 4.65 |
| **P1C1-TM** | 2.72 ± 0.123 | 1.39 ± 0.0577 | 5.1 ± 0.0980 |

**Supplementary Fig. 2.** Binding kinetics measurements by surface plasmon resonance. Soluble recombinant $p53_{125\text{-}134}$/A24 pMHCs was flowed over **(A)** P1C1gl and affinity matured clones **(B)** 1E11, **(C)** 2E3 and **(D)** 1G7 captured on a sensor chip, at a range of concentrations between 200nM to 2nM. Sensorgrams were referenced against a control lane (no immobilized anti-human IgG) and fitted globally to a 1:1 Langmuir binding model. **(E)** Kinetic constants ($k_a$ and $k_d$) and affinity ($K_D$) were determined using the BIAevaluation software and the averages and SEM of 2 to 4 separate measurements were subsequently calculated and tabulated.

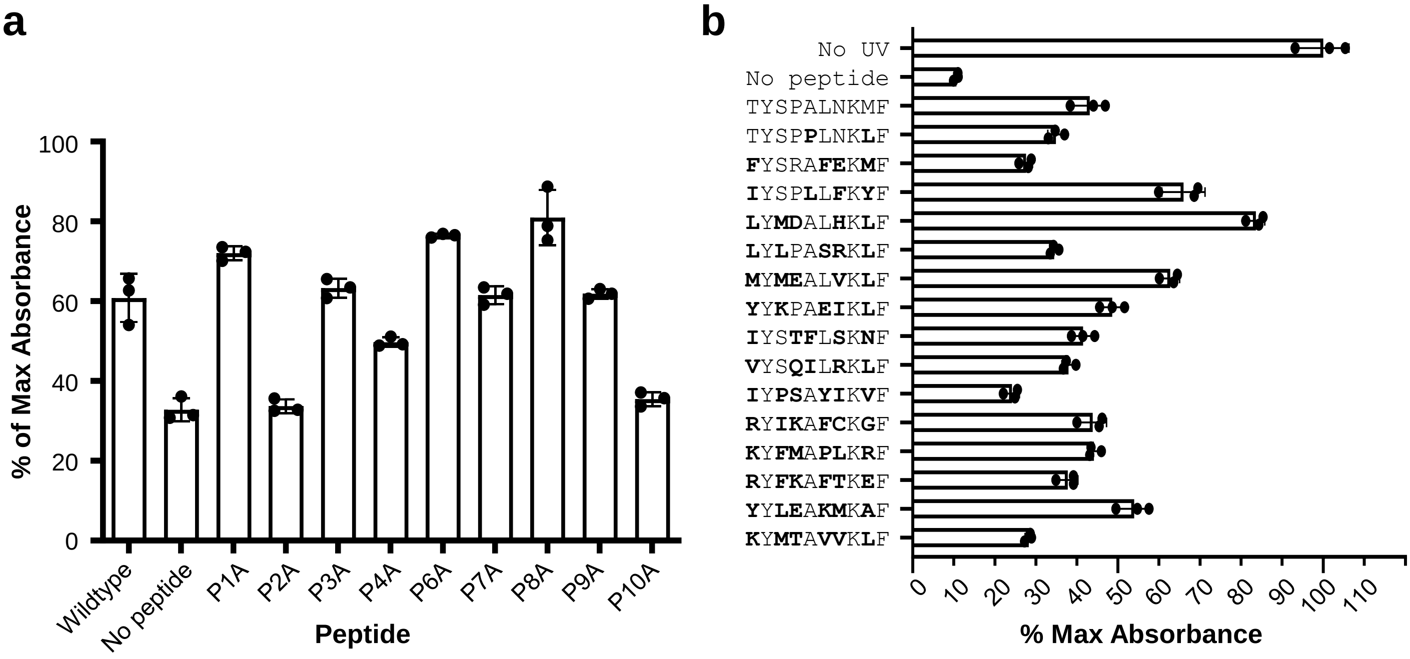


**Supplementary Fig. 3.** Peptide analogues pMHC stability ELISA. UV-cleavable peptide HLA-A24 pMHC was subjected to UV irradiation in the presence of **(A)** Single amino acid alanine mutants of the $p53_{125\text{-}134}$ peptide or **(B)** potential cross-binding peptides or in the absence of any peptides. The stability of the peptide-exchanged pMHC was assessed by ELISA. UV-exchanged pMHC were captured by immobilized neutravidin and the levels of intact pMHC were assessed by probing for the presence of beta-2-microglobulin. Intact UV-cleavable pMHC not subjected to any UV was used as a positive control (max absorbance) and all results were expressed as a percentage of the no UV control. Data are means of triplicates ±SEM.

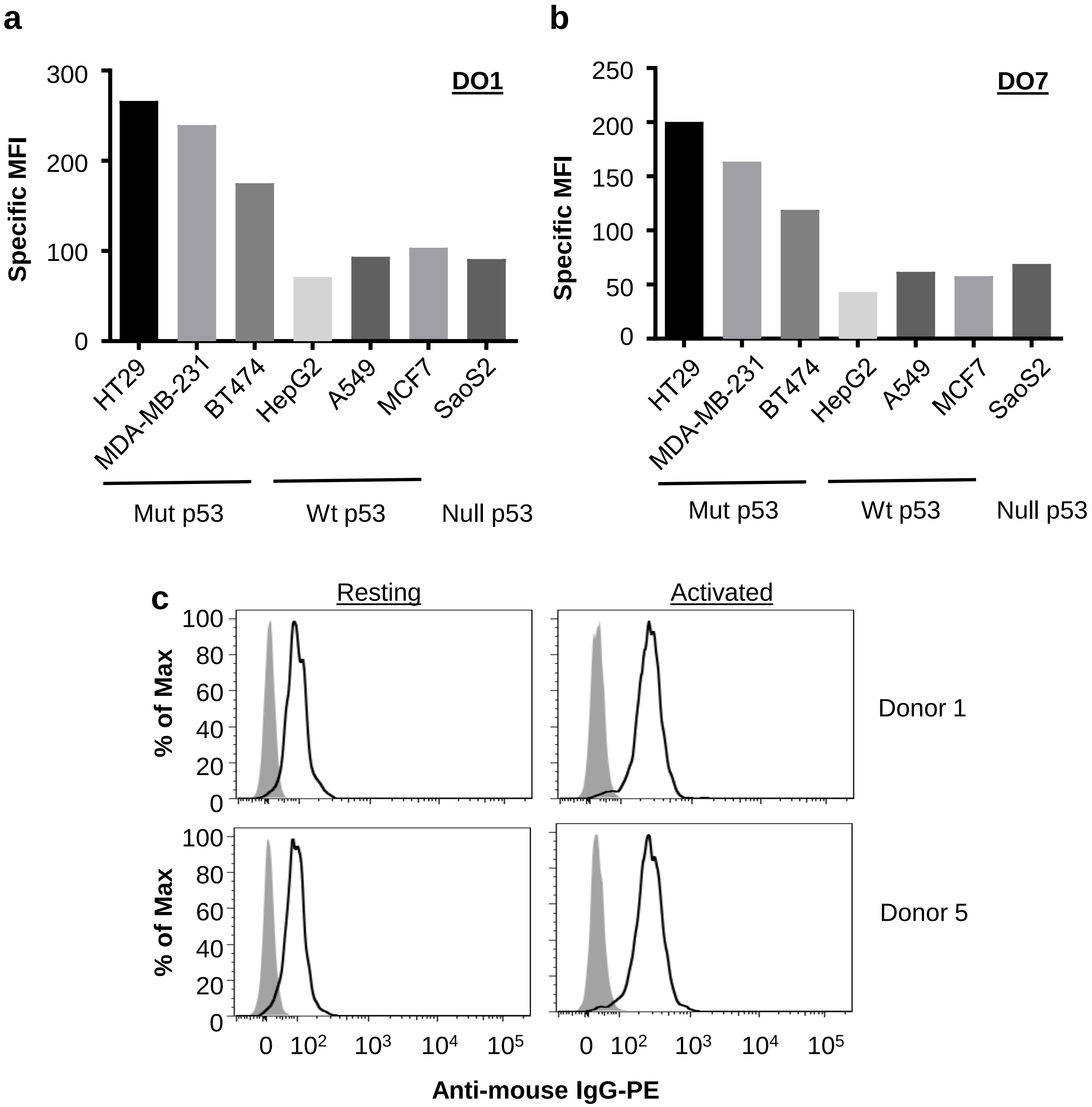


**Supplementary Fig. 4.** Intracellular staining of p53. Comparison of intracellular levels of p53 proteins between cell lines expressing mutant p53 (HT29, MDA-MB-231 and BT474), wild-type p53 (HepG2, A549 and MCF-7) and no p53 (SaoS2) was done by the intracellular staining of intracellular p53 proteins using the **(a)** DO1 and **(b)** DO7 antibodies respectively. Data is representative of two independent experiments. **(c)** Resting and activated purified T cells from healthy donors were stained intracellularly with the anti-p53 DO7 antibody. Levels of intracellular p53 in T cells increased upon activation.

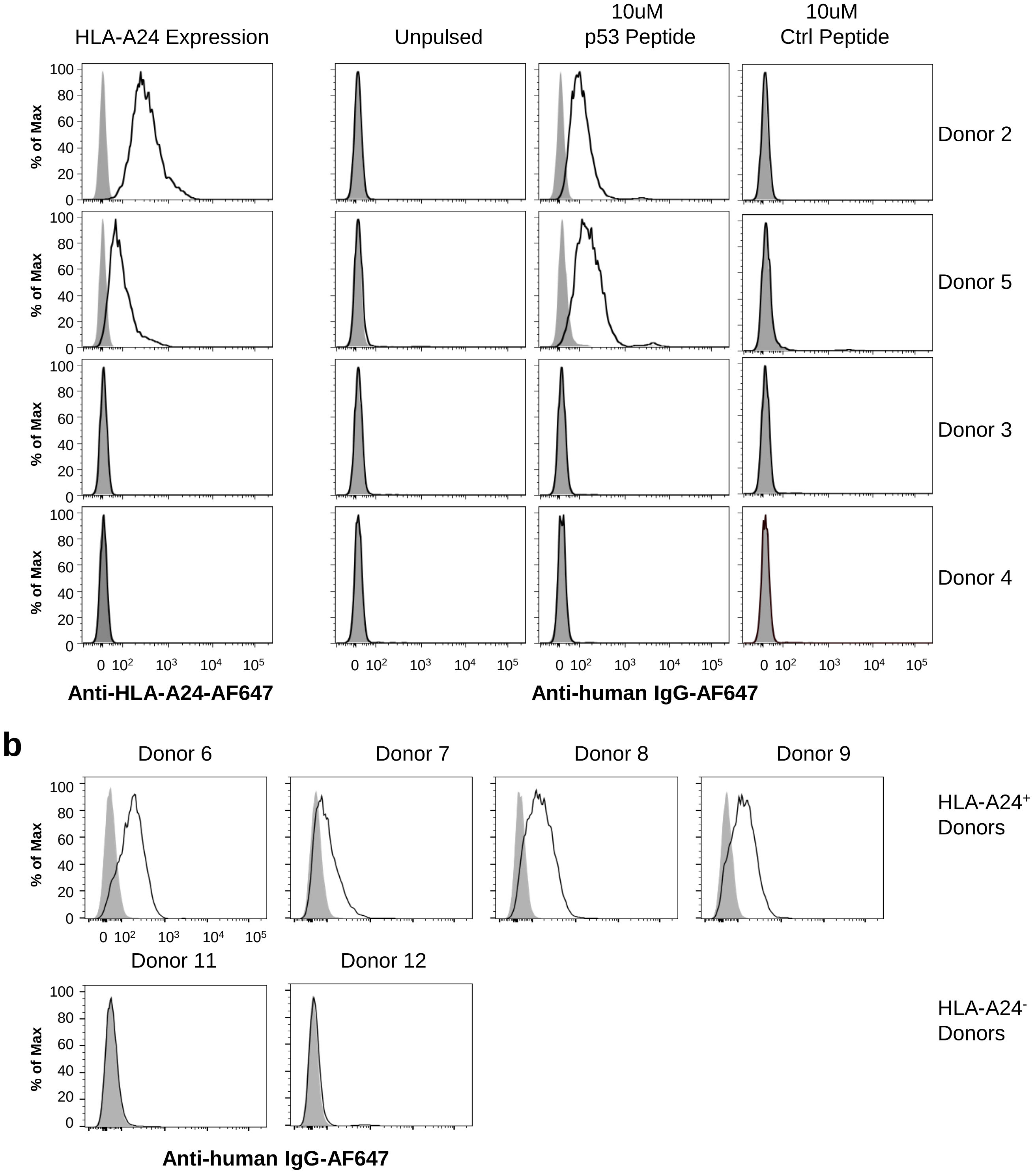


**Supplementary Fig. 5.** Detection of p53$_{125\text{-}134}$/A24 pMHC on healthy immune cells by P1C1TM. **(a)** Total peripheral blood mononuclear cells from healthy HLA-A24$^{+}$ and HLA-A24$^{-}$ donors were stained with P1C1TM with or without exogenous peptides pulsed. Staining of P1C1TM was observed only with HLA-A24$^{+}$ PBMCs after pulsing with the p53$_{125\text{-}134}$ peptide. No staining was observed in HLA-A24$^{-}$ PBMCs. **(b)** Purified T cells from healthy donors were activated and stained with P1C1TM. Only activated HLA-A24$^{+}$ T cells were stained positive by P1C1TM.

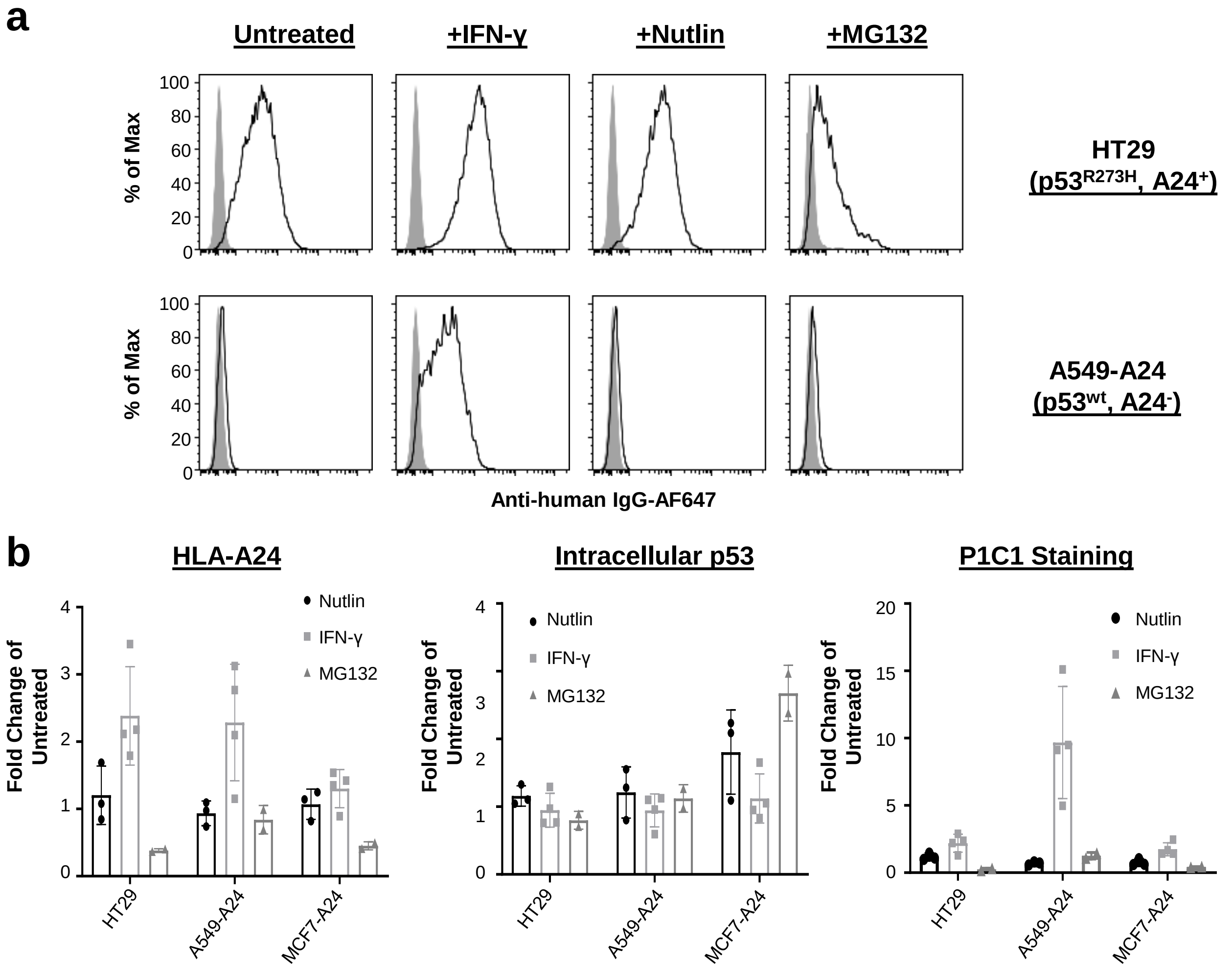


**Supplementary Fig. 6.** Effects of interferon-γ (IFN-γ), nutlin and MG132 on presentation of $p53_{125\text{-}134}$/A24 pMHCs in treated cell lines. **(a)** HT29 cells expressing mutant p53 and A549-A24 cells expressing wildtype p53 were treated with the respective molecules and stained with P1C1TM to monitor $p53_{125\text{-}134}$/A24 presentation. IFNγ treatment increased P1C1TM staining on both cell lines whilst MG132 significantly reduced P1C1TM in treated HT29 cells. **(b)** Effect of the treatments on levels of HLA-A24, intracellular p53 and presentation of p53125-134/A24 pMHCs in HT29, A549-A24 and MCF7-A24 cells.

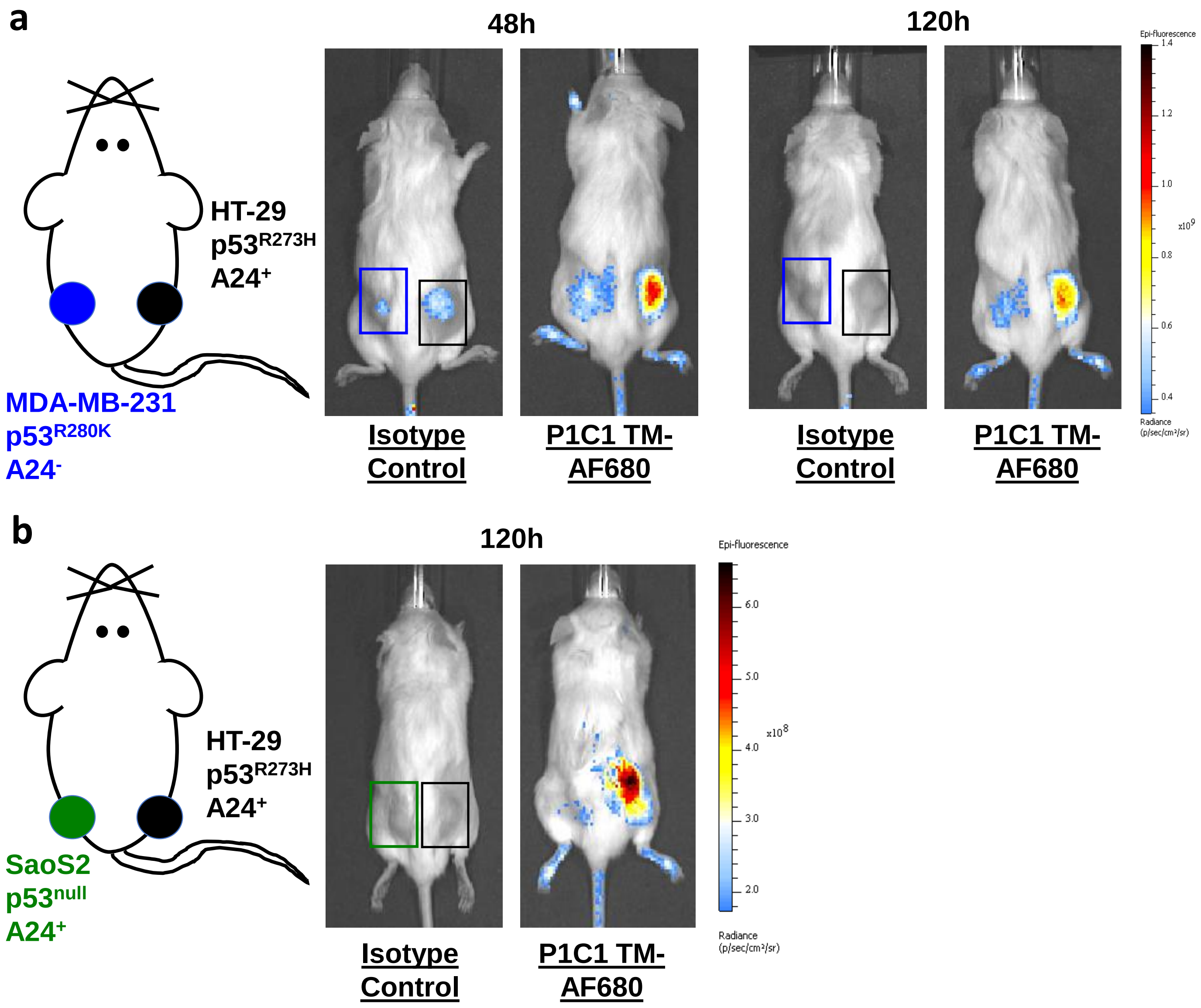


**Supplementary Fig. 7.** In vivo specificity of TCR-like antibody P1C1TM. NSG mice were injected subcutaneously with either **(A)** HT29 (HLA-A24$^{pos}$, p53$^{R273H}$) and MDA-MB-231 (HLA-A24$^{pos}$, p53$^{R280K}$) or **(B)** HT29 (HLA-A24$^{pos}$, p53$^{R273H}$) and SaoS2 cells (HLA-A24$^{pos}$, p53$^{null}$). After tumor sizes were ≥ 100mm$^3$, 50µg of P1C1TM conjugated with the Alexa Fluor 680 dye was injected IV through the tail vein. Binding of the labeled antibody to tumors was detected using the IVIS® imaging system 48 and/or 120 hours post antibody administration.

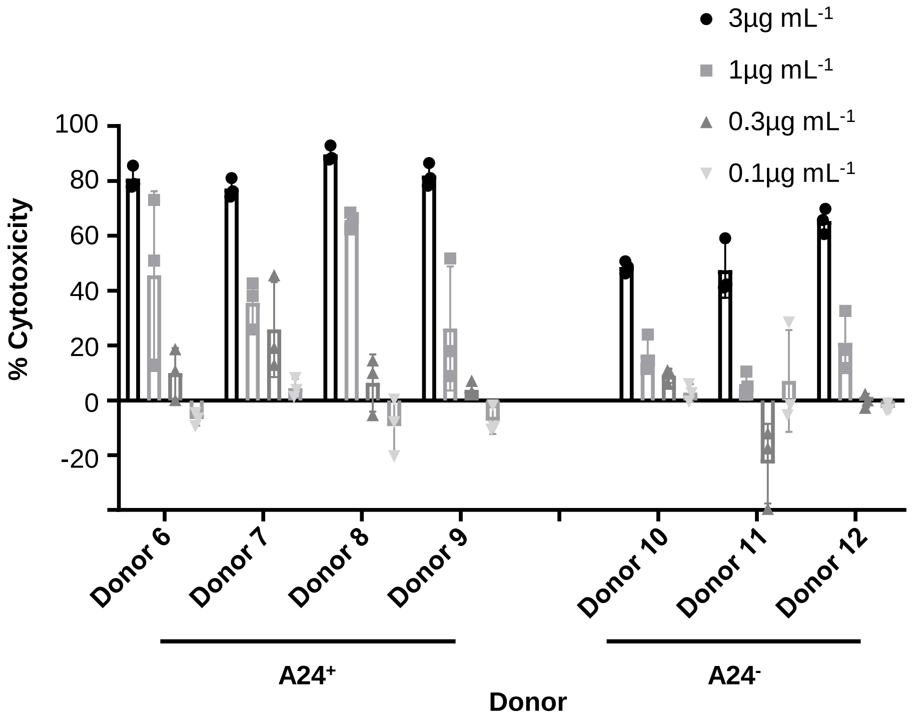


**Supplementary Fig. 8.** Effect of P1C1TM-PNU on activated T cells. Activated purified T cells from healthy donors were incubated with a range of concentrations of P1C1TM-PNU. After 2 days of incubation, viability was measure by alamarBlue cell viability assay and %cytotoxicity was calculated with respect to the untreated cells.

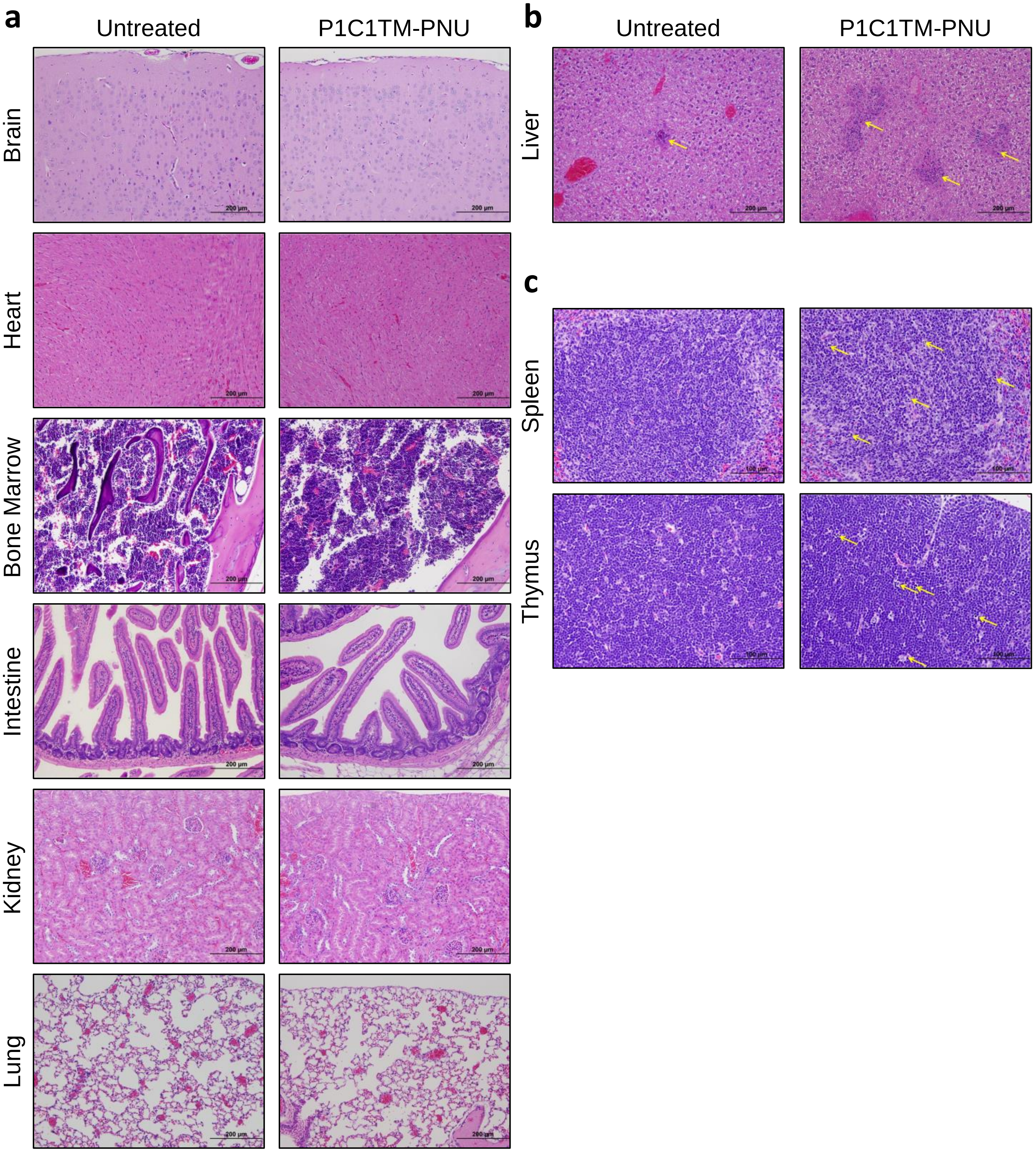


**Supplementary Fig. 9.** Toxicity analysis of P1C1TM-PNU in HLA-A24 transgenic mice. Mice were treated with 1mg $kg^{-1}$ of P1C1TM-PNU and monitored. Mice were sacrificed after 20 days and an examination of hematoxylin and eosin stained tissues was done. Representative photomicrographs are shown. **(A)** Several tissues exhibited no significant differences between untreated (left) and P1C1TM-PNU treated (right). **(B)** Infiltrates of polymorphonuclear, mononuclear cells (as indicated by arrows) were observed in the livers of both untreated and treated mice while **(C)** mild lympholysis (as indicated by arrows) was observed only in spleen and thymus of treated mice.

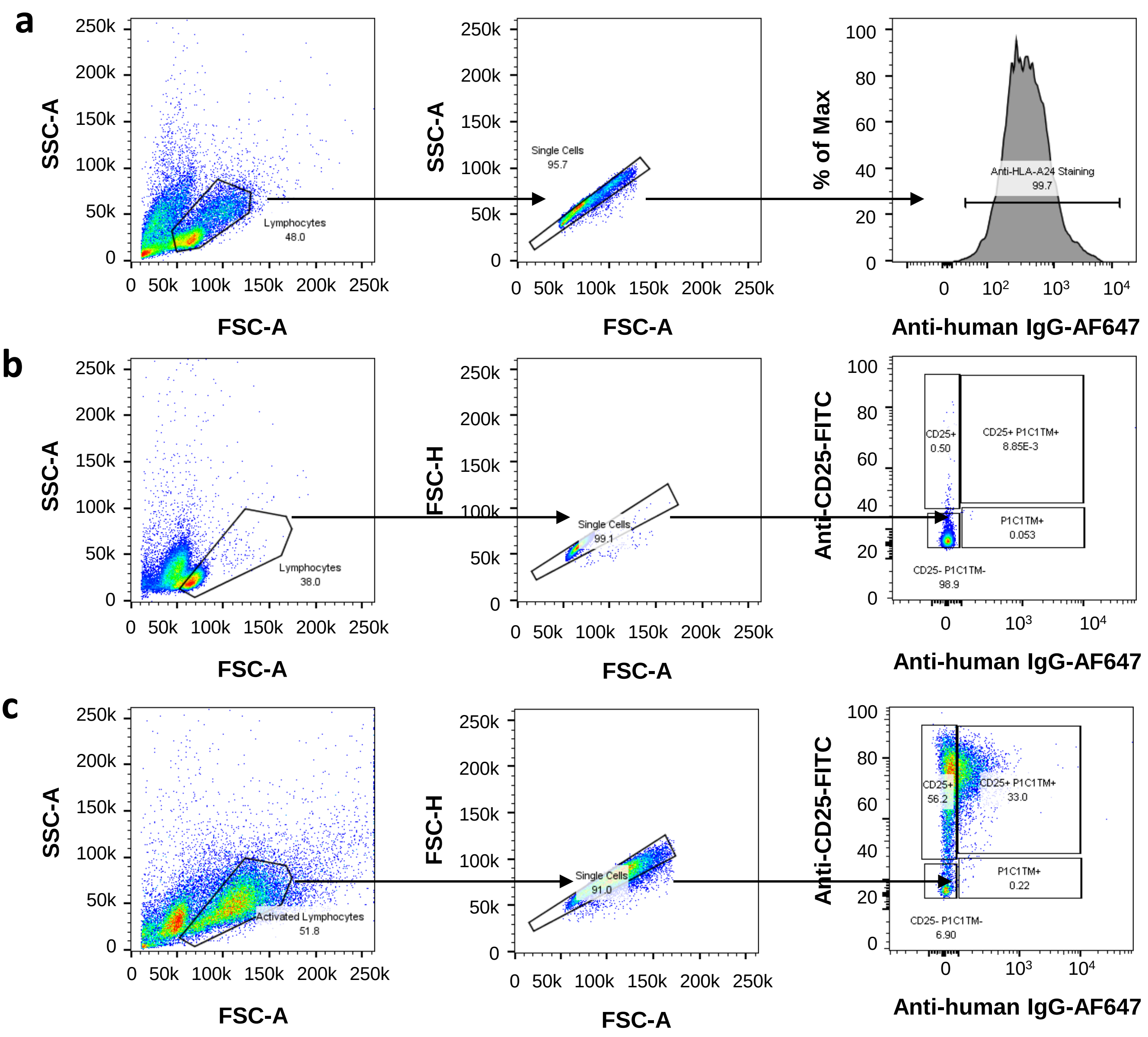


**Supplementary Fig. 10.** Gating strategy used for immune cell staining. (A) A representative plot for the gating strategy for PBMCs assessed for antibody binding in Fig 4d are shown. PBMCs were identified by FSC-A and SSC-A before doublet discrimination by FSC-H and FSC-A. Singlet cells were then examined for antibody staining. Representative plots for the gating strategy of (B) unactivated purified T cells and (C) activated purified T cells examined for antibody binding in Fig. 4e and Supplementary Figs 4 and 5 are shown.
