# Supplementary material for: Targeting mutant p53-expressing tumours with a T cell receptor-like antibody specific for a wild-type antigen
Source: Nat Commun. 2019 Nov 26;10:5382. doi: 10.1038/s41467-019-13305-z (PMC6879612; doi:10.1038/s41467-019-13305-z)
Supplement: Supplementary file 3 — Reporting Summary [file 41467_2019_13305_MOESM3_ESM.pdf]

natureresearch

Corresponding author(s): Cheng-I Wang

Last updated by author(s): Oct 9, 2019

Reporting Summary

Nature Research wishes to improve the reproducibility of the work that we publish. This form provides structure for consistency and transparency in reporting. For further information on Nature Research policies, see Authors & Referees and the Editorial Policy Checklist .

Statistics

For all statistical analyses, confirm that the following items are present in the figure legend, table legend, main text, or Methods section.

n/a	Confirmed	
☐	☒	The exact sample size (*n*) for each experimental group/condition, given as a discrete number and unit of measurement
☐	☒	A statement on whether measurements were taken from distinct samples or whether the same sample was measured repeatedly
☐	☒	The statistical test(s) used AND whether they are one- or two-sided<br>*Only common tests should be described solely by name; describe more complex techniques in the Methods section.*
☒	☐	A description of all covariates tested
☐	☒	A description of any assumptions or corrections, such as tests of normality and adjustment for multiple comparisons
☒	☐	A full description of the statistical parameters including central tendency (e.g. means) or other basic estimates (e.g. regression coefficient) AND variation (e.g. standard deviation) or associated estimates of uncertainty (e.g. confidence intervals)
☐	☒	For null hypothesis testing, the test statistic (e.g. *F*, *t*, *r*) with confidence intervals, effect sizes, degrees of freedom and *P* value noted<br>*Give P values as exact values whenever suitable.*
☒	☐	For Bayesian analysis, information on the choice of priors and Markov chain Monte Carlo settings
☒	☐	For hierarchical and complex designs, identification of the appropriate level for tests and full reporting of outcomes
☒	☐	Estimates of effect sizes (e.g. Cohen's *d*, Pearson's *r*), indicating how they were calculated

Our web collection on statistics for biologists contains articles on many of the points above.

Software and code

Policy information about availability of computer code

Data collection	Biacore T200 software was used to measure the on and off rate of binding of the antibodies.
Data analysis	Biacore Insight Evaluation Software was used for analyzing the binding kinetics of the antibodies.

For manuscripts utilizing custom algorithms or software that are central to the research but not yet described in published literature, software must be made available to editors/reviewers. We strongly encourage code deposition in a community repository (e.g. GitHub). See the Nature Research guidelines for submitting code & software for further information.

Data

Policy information about availability of data

All manuscripts must include a data availability statement. This statement should provide the following information, where applicable:

- Accession codes, unique identifiers, or web links for publicly available datasets
- A list of figures that have associated raw data
- A description of any restrictions on data availability

The source data underlying Figs. 1-6 and Supplementary Figs 3, 4, 6 and 8 are provided as a Source Data file. All the other data supporting the findings of this study are available within the article and its supplementary information files and from the corresponding author upon reasonable request. A reporting summary for this article is available as a Supplementary Information file.

Field-specific reporting

Please select the one below that is the best fit for your research. If you are not sure, read the appropriate sections before making your selection.

Life sciences study design

All studies must disclose on these points even when the disclosure is negative.

Sample size	No sample size calculation was performed. The number of repeats in sample replicates and experimental replicates are provided in the methods and figure legends. Antibody binding was tested in three cell lines expressing both wild type and three cell lines expressing mutant p53.
Data exclusions	A mouse in the P1C1TM-PNU treated group in the study described in Fig 7a was excluded due to anomalous growth rate observed.
Replication	Binding assays by ELISA and with cell lines by flow cytometry, ADCC and cytotoxicity assays were repeated at least twice.
Randomization	Mice subcutaneously implanted with xenografts were randomly assigned into the various treatment and control groups.
Blinding	n/a

Reporting for specific materials, systems and methods

We require information from authors about some types of materials, experimental systems and methods used in many studies. Here, indicate whether each material, system or method listed is relevant to your study. If you are not sure if a list item applies to your research, read the appropriate section before selecting a response.

Materials & experimental systems

n/a	Involved in the study	
☐	☒	Antibodies
☐	☒	Eukaryotic cell lines
☒	☐	Palaeontology
☐	☒	Animals and other organisms
☒	☐	Human research participants
☒	☐	Clinical data

Methods

n/a	Involved in the study	
☒	☐	ChIP-seq
☐	☒	Flow cytometry
☒	☐	MRI-based neuroimaging

Antibodies

Antibodies used	P1C1, P1C1gl, P1C1-1G7, P1C1-2E3, P1C1-1E11, P1C1TM (All produced in house)<br>Peroxidase AffiniPure goat anti-human IgG F(ab')2 fragment specific (Jackson Immunoresearch #109-036-097), peroxidase AffiniPure goat anti-human IgG, Fcγ fragment specifc (Jackson Immunoreseach #109-035-097), mouse anti-human beta-2-microglobulin antibody (BD Pharmingen, clone TU99), Peroxidase AffiniPure goat anti-mouse IgG (H+L) (Jackson Immunoresearch #115-034-146), goat anti-human IgG (H+L) cross-adsorbed secondary antibody, AF647 conjugate (Life Technologies, #A-21445), anti-HLA-A24 (Human) mAb-AF647 (MBL International #0208-A64), p53 monoclonal antibody clone DO-1 unconjugated (Life Technologies #MA5-12571), p53 monoclonal antibody clone DO-7 (#MA5-12557), goat anti-mouse IgG (H+L) cross-adsorbed secondary antibody, PE conjugate (Life Technologies #P-852), mouse anti-human IgG (CH2 domain) secondary antibody (ThermoFisher, #MA5-16929), anti-human IgG Fc-MMAE antibody with cleaveable linker (Moradec LLC, #AH-102AE), anti-human IgG Fc-PNU159682 antibody with cleaveable linker (Moradec LLC, #AH-102PN), anti-human IgG Fc-PBD antibody with cleaveable linker (Moradec LLC, #AH-106PB), anti-human IgG Fc-Amanitin antibody with non-cleaveable linker (Moradec LLC, #AH-205AM)
Validation	Validation of commercial antibodies were provided by manufacturers.

Eukaryotic cell lines

Policy information about cell lines

Cell line source(s)	The cell lines MDA-MB-231, A549, BT474, MCF-7 and Hep-G2 were purchased from the American Type Culture Collection (ATCC). SaoS2 and HT29 which were kind gifts from colleagues,
Authentication	Identity of cell lines purchased from ATCC were verified by the specification sheets provided. Whilst SaoS2 and HT29 were verified by morphology and not by any other methods.
Mycoplasma contamination	Mycoplasma contamination was done routinely in the lab.
Commonly misidentified lines (See ICLAC register)	n/a

Animals and other organisms

Policy information about studies involving animals; ARRIVE guidelines recommended for reporting animal research

Laboratory animals	Six to eight weeks old NOD-scid IL2rgnull (NSG) mice were used for the in vivo imaging and efficacy studies. For the cross reactivity and off-target toxicity studies, six to eight week old CB6F1-Tg(HLA-A*2402/H2-Kb)A24.01 (HLA-A24 transgenic) mice were used.
Wild animals	n/a
Field-collected samples	n/a
Ethics oversight	All experiments involving animals were performed in accordance with guidelines approved by the Institutional Animal Care and Use Committee of the Biological Resource Center (BRC), Agency for Science, Technology and Research (A*STAR),

Note that full information on the approval of the study protocol must also be provided in the manuscript.

Flow Cytometry

Plots

Confirm that:

- [x] The axis labels state the marker and fluorochrome used (e.g. CD4-FITC).
- [x] The axis scales are clearly visible. Include numbers along axes only for bottom left plot of group (a 'group' is an analysis of identical markers).
- [x] All plots are contour plots with outliers or pseudocolor plots.
- [x] A numerical value for number of cells or percentage (with statistics) is provided.

Methodology

Sample preparation	For cell binding assays, cell lines were harvested from culture, washed and stained with the antibodies at room temperature for 30mins. Cells were then washed twice and stained with fluorescently labeled secondary antibodies at room temperature for 30 mins, protected from light. All washes were done in staining buffer (PBS with 1% BSA).<br>PBMCs from healthy donors were isolated from buffy coats using a Ficoll-Hypaque density gradient centrifugation. T cells were then purified from PBMCs by magnetic separation using a pan T cell isolation kit (Miltenyi Biotec) and activation was done with T cell TransAct (Miltenyi Biotec). T cells were then stained similarly as the cell lines described above.<br>For pulsed cell staining assay, harvested cells were pre-incubated with peptides for 1hr at 37deg prior to staining as mentioned above.<br>For intracellular staining assays, harvested cells were first fixed and permeabilized using the Fixation/Permeabilization solution (BD Biosciences) for 15 mins on ice, protected from light. Cells were then washed in Perm/Wash buffer (BD Biosciences) 4 times before staining with antibodies at room temperature for 30 mins. Secondary antibody staining was similarly done as described above.<br>For internalization assays, harvested cells were stained with antibodies conjugated with pHrodo Red dyes at 37deg or on ice. At indicated time points, cells were quenched in ice cold staining buffer (PBS with 1% BSA) and acquired.
Instrument	BD FACSVerse
Software	FlowJo Ver. 8 and Ver. 10
Cell population abundance	At least 10,000 events were acquired and recorded for all samples.
Gating strategy	For T cell staining assays, T cells were gated using FSC-A and SSC-A followed by single cell discrimination using FSC-A and FSC-H. Activated T cells were defined as CD-25 high and cells positive for anti-human AF647 signal were defined as positive for antibody binding.

- [x] Tick this box to confirm that a figure exemplifying the gating strategy is provided in the Supplementary Information.
